# [Corrigendum] Resistin aggravates the expression of proinflammatory cytokines in cerulein-stimulated AR42J pancreatic acinar cells

**DOI:** 10.3892/mmr.2026.13960

**Published:** 2026-07-08

**Authors:** Chong-Yi Jiang, Wei Wang

Oncol Lett 15: 502–506, 2017; DOI: 10.3892/mmr.2016.6027

Following the publication of the above paper, the authors contacted the Editorial Office to explain that the western blots included in Fig. 3 on p. 504 had inadvertently been included in error. Given the time that has elapsed since the publication of this paper, the authors no longer had access to their original data, and so they have repeated these experiments, and the revised version of this figure is presented below. Note that the results obtained in these experiments are broadly similar to the published results, and the overall scientific conclusions reported in the article are not affected by repeating these experiments. The authors are grateful to the Editor of *Oncology Letters* for allowing them the opportunity to publish this Corrigendum, and all the authors agree with its publication.

## Figures and Tables

**Figure 3.**
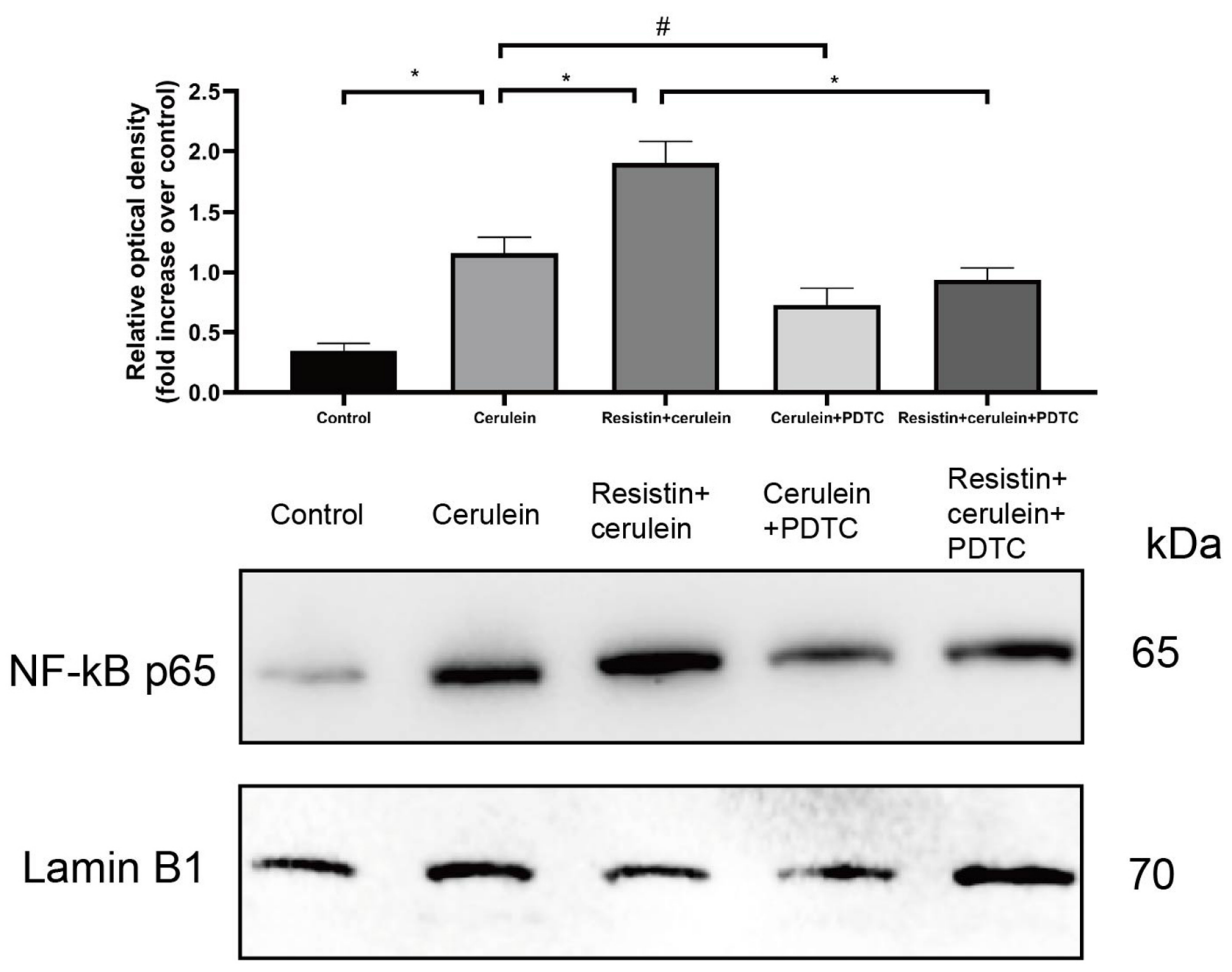
Western blot analysis of protein levels of the NF-κB p65 subunit in the nuclear fraction of AR42J cells. AR42J cells were incubated with or without NF-κB inhibitor PDTC for 2 h, followed by stimulation with cerulein or cerulein+resistin. Subsequently, cells were subjected to nuclear extraction to detect protein levels of the NF-κB p65 subunit. Protein expression was normalized to lamin B. The data are presented as the mean ± standard deviation of three independent experiments, each performed in duplicate. *P<0.01 and #P<0.05. NF-κB, nuclear factor-κB; PDTC, pyrrolidine dithiocarbamate.

